# Sex-Related Differences in the Pathophysiology, Cardiac Imaging, and Clinical Outcomes of Aortic Stenosis: A Narrative Review

**DOI:** 10.3390/jcm13216359

**Published:** 2024-10-24

**Authors:** Abdellaziz Dahou, Vikky Awasthi, Meriem Bkhache, Merouane Djellal, Xiaofeng Yang, Hong Wang, Rihab Bouchareb

**Affiliations:** 1 Cardiology Division, Department of Medicine, Massachusetts General Hospital, 55 Fruit Street, Boston, MA 02114, USA; adahou1@mgh.harvard.edu; 2 Lewis Katz School of Medicine at Temple University, 3500 North Broad Street, Philadelphia, PA 19140, USA; 3 Center for Metabolic Disease Research (CMDR), Department of Cardiovascular Sciences, Lewis Katz School of Medicine at Temple University, 3500 North Broad Street, Philadelphia, PA 19140, USA

**Keywords:** aortic valve, sex, calcification, echocardiography

## Abstract

Aortic stenosis (AS) is the most common valvular heart disease in developed countries, and its prevalence is higher in older patients. Clinical studies have shown gender disparity in the pathogenesis and the progression of aortic stenosis. This disparity has led to several overwhelming questions regarding its impact on the clinical outcomes and treatment of the disease and the requirement of personalized sex-specific approaches for its management. Indeed, aortic stenosis differs in the pathophysiological response to pressure overload created by the stenosis in women compared to men, which would translate into differences in cardiac remodeling and clinical outcomes. Several studies have focused on understanding the differences regarding disease progression according to biological gender and have found that sex hormones play a crucial role. Sex hormones affect many metabolic processes, thus activating crucial cell signaling and energy metabolism through mitochondrial activity. Yet, there is still a significant gap in knowledge on how biological sex influences the pathophysiology of AS. In this review, we have discussed studies that point to the role of sex-related physiological differences in the molecular pathways and the clinical presentation of the disease and outcome in women and men. We used the format of narrative review to review and summarize the body of literature without being systematic but with taking great care of considering the most impactful data available to date on the topic, especially randomized trials, metanalyses, and prospective studies and registries when available, as well as experimental studies with rigorous methodological approaches regarding the basic mechanisms and pathophysiology of the disease in women compared to men. The opinion of the authors on a particular issue or finding was expressed when appropriate for clarification.

## 1. Introduction

Aortic stenosis (AS) is the most prevalent valvular heart disease in developed countries, and the only available treatment currently is surgical or transcatheter aortic valve replacement [1,2]. The initial phase of the disease involves the stiffening of aortic valve leaflets, known as the sclerotic phase, caused by excessive deposition of extracellular matrix (ECM) [3], which will then eventually evolve into stenosis, characterized by inflammation, endothelial dysfunction, lipid deposition, and the accumulation of calcium deposits that distort the valve’s structure, accompanied by the development of neovascularization [4,5]. This process initially manifests with subtle or no clinical symptoms [6]. The transition from sclerosis to stenosis occurs in about 10–15% of individuals within a period of 2 to 5 years, according to some data [7], but may vary from one patient to another with potential differences between men and women. Patients with more advanced valve obstruction (moderate vs. mild stenosis) typically undergo a more rapid progression toward severe stenosis, requiring valve replacement [8,9].

**Figure 1:**
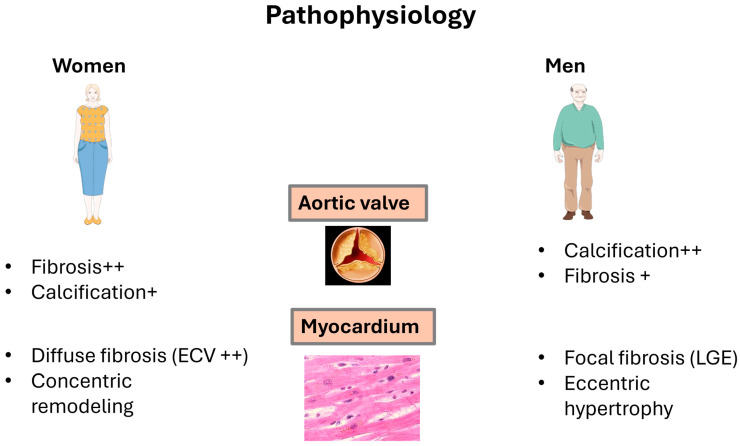
The nature of aortic stenosis differs between men and women. In women, it is marked by increased fibrosis and decreased calcification, while in men, the valve calcification is more severe and more dominant than fibrosis. Furthermore, in women, the progression of aortic stenosis is marked by concentric remodeling of LV with diffuse fibrosis, represented by increased fraction of extracellular volume (ECV), while in men, it presents more eccentric hypertrophy and focal fibrosis, represented by the presence of late gadolinium enhancement (LGE).

Increasing evidence is showing differences in the mechanisms of valvular remodeling and differences in the severity of calcification and fibrosis between men and women [10] (Figure 1), which, in the end, translates into differences in clinical presentation and outcome [11,12]. This highlights the necessity for dedicated sex-specific investigations to understand the underlying mechanisms behind this dimorphism better and enhance treatment effectiveness and outcomes.

## 2. Biological Sex Hormones and Aortic Valve Cell Calcification

### 2.1. The Link between Sex Hormones and AS

Aortic valve stenosis has been linked to gender differences, as observed in various epidemiological studies. The prevalence of aortic stenosis is higher in men as compared to women [13]. The disparities in disease outcomes between genders are thought to stem from varying levels of sex steroid hormones, particularly estrogen and testosterone. This potential link between sex hormone signaling and the development and progression of aortic stenosis was evident from studies in mice, which demonstrated the upregulation of genes associated with aortic valve mineralization in males compared to females [14]. In humans, a cohort study conducted in middle-aged and older Finnish men found a positive association between elevated serum testosterone levels and the risk of developing (AS) [15]. Eildermann et al. investigated androgen receptor (AR) expression in human heart tissue. Their findings showed upregulated AR mRNA levels in patients with aortic stenosis compared to healthy controls [16]. 

**Figure 2:**
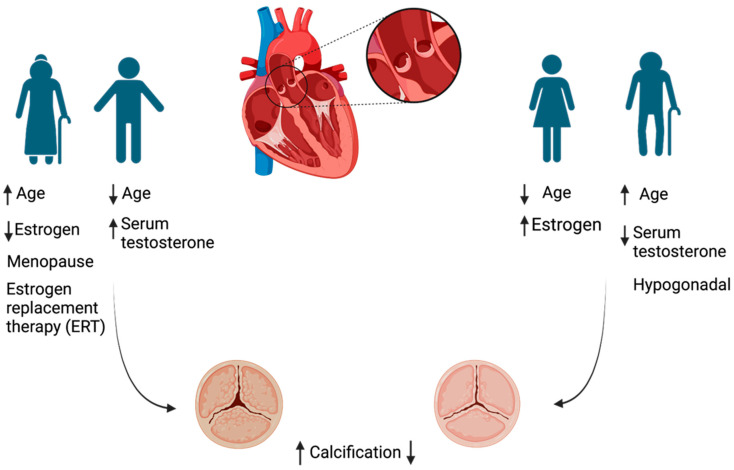
Aortic stenosis in men is characterized by more calcification, while in women, it is characterized by more fibrosis. For both men and women, the incidence of AS increases with age. However, sex hormones significantly impact the progression of AS in men and women. Men develop AS at a younger age compared to women, while the incidence of AS decreases in hypogonadal males. In women, estrogen protects from AS, and when its level decreases with an increase in age and after menopause, the incidence of the disease increases. Furthermore, estrogen replacement therapy (ERT) decreases AS in women.

Similarly, the role of estrogen in aortic stenosis development is evident from clinical studies, which have reported a lower incidence of cardiovascular disease (CVD), including aortic stenosis, in premenopausal women compared to age-matched males. This disparity narrows and even reverses after menopause, suggesting a protective role of estrogen during reproductive years [17,18] (Figure 2).

Sex hormones undoubtedly influence cardiovascular health. Studies utilizing gonadectomized animal models continue to demonstrate sex-related dimorphic disease progression [19]. In an exciting study [1], the hormone effect was well demonstrated in the development of AS using castrated and ovariectomized mice supplemented with respective hormones. The highest hemodynamic progression of AS was observed in intact male mice, which significantly decreased following castration. Interestingly, testosterone supplementation in castrated males partially restored hemodynamic progression, suggesting a dose-dependent effect of androgens on AS severity. This observation aligns with the reported four-fold increase in calcification deposits in intact mice compared to castrated mice. Conversely, the study suggests a minimal role for estrogen in the murine aortic valve pathophysiology. While intact female mice (IF) displayed downregulation of genes, Alkaline Phosphatase and Angiotensin II receptor type 1 associated with valve homeostasis compared to ovariectomized females (OF), supplementation with 17β-estradiol (OFE) did not significantly alter this expression pattern [1].

### 2.2. Molecular Mechanisms and Sex Hormones in AS

Mechanisms underlying aortic stenosis progression under the influence of sex hormones in humans are still elusive. Sex steroids exert their pleiotropic effects through interactions with distinct receptor subtypes. Estrogen predominantly binds to nuclear estrogen receptors (ERs), including ERα and ERβ. Additionally, it can activate the G protein-coupled estrogen receptor (GPER) [20]. Male sex hormones, testosterone, and its metabolite DHT exert their actions via androgen receptors (ARs) [21]. These receptors exhibit both cytoplasmic and membrane localization, mediating diverse genomic and non-genomic effects involving the activation of multiple intracellular signaling cascades and influencing intracellular calcium (Ca^2^⁺) concentration, nitric oxide (NO) synthesis, protein kinase C (PKC), and mitogen-activated protein kinase (MAPK) pathway [22]. These pathways have an instrumental role in the development of aortic stenosis. MAPK signaling is involved in AS [23], while PKC activation inhibits endothelial NO synthase [23], and NO activates NOTCH signaling through S-nitrosylation of USP9X24, preventing AS. 

**Figure 3:**
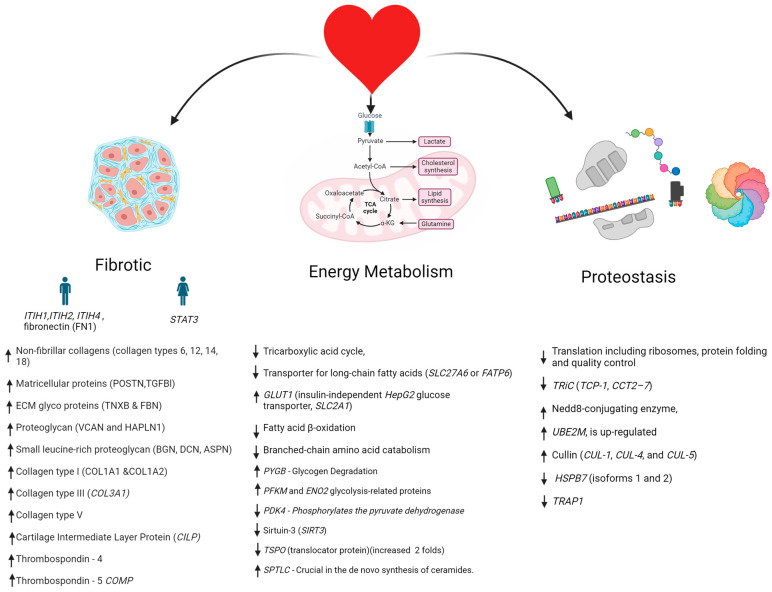
Gender-related protein markers for aortic stenosis. The mechanism of aortic stenosis progression is illustrated with an increase in several molecules related to fibrotic events in the aortic valve, changes in the metabolic status of cells, and proteostasis. During fibrosis, the expression of ITIH1, 2, and 4 and the fibronectin gene increases specifically, while in women, the transcriptional regulator STAT3 increases. Additionally, all genes listed with the upward arrow increase in both biological genders, while the arrows pointing downwards show a decrease in gene expression.

A study showed that culturing valve interstitial cells (VICs) in either a pro-inflammatory or quiescent state did not significantly alter calcium deposition. However, treatment with testosterone (concentration- and duration-dependent) significantly enhanced calcification in both VICs (~16-fold increase) and valve smooth muscle cells (VSMCs) (~5-fold increase) compared to controls, whereas estrogen treatment had no observable effect. Similarly, proteomic profiling of male aortic stenosis patients revealed an upregulation of proteins associated with the extracellular matrix, predominantly proteins of the ITIH family (ITIH1 and ITIH2), which are ECM stabilizers. Additionally, Fibronectin1 and serpine E2 are elevated. Upregulation of serpineE2 leads to increased fibrosis. In contrast, female patients displayed a marked reduction in STAT3, crucial in cardiac fibrosis and collagen synthesis. Several genes upregulated in both genders include proteoglycan small leucine-rich proteoglycan, collagen type I, III, and V, Cartilage Intermediate Layer Protein (*CILP*), and thrombospondin 4 and 5. Further, there is downregulation of proteins involved in cellular energy metabolism like tricarboxylic acid cycle, transporter for long-chain fatty acids (*SLC27A6*), *GLUT1*, fatty acid β-oxidation, branched-chain amino acid catabolism *PYGB*, and glycogen degradation glycolysis-related proteins *PDK4* and *SPTLC*—crucial in the de novo synthesis of ceramides. The proteostasis genes that are upregulated include related genes like Nedd8-conjugating enzyme, *UBE2M*, and *HSPB7* (isoforms 1 and 2), while the *TRiC* and *TRAP1* genes protecting the heart from hypertrophy [24] are downregulated (Figure 3). 

A study by Masjedi et al. [25] showed the presence of sex-related differences in early osteogenic differentiation of the aortic valves at the cellular level. The in vitro study showed an increased proliferation rate in female rat aortic valvular interstitial cells (RAVICS) and porcine aortic valvular interstitial cells (PAVICS) compared to males. In addition, they showed an elevated matrix remodeling: male RAVICS had higher collagen I, Glycosaminoglycan (GAG), and activated matrix metalloproteinase MMP-2 after 15 days of culture in osteogenic media. Moreover, they found an (early osteogenic marker) ALP-positive cells higher in male RAVICS than in females. Male PAVICs have higher calcified nodules after 15 days of culture in osteogenic media. The study also showed an increase in cell proliferation rate in female RAVICS after β-estradiol treatment, while males’ proliferation was independent of the treatment amount [25].

### 2.3. The Role of Testosterone

Testosterone plays a role in calcium homeostasis and can elevate serum calcium levels. Studies in testosterone-deprived male rats have shown that testosterone replacement therapy can attenuate intracellular calcium dyshomeostasis within the heart [26]. Androgens promote calcification [27] and also exert an effect on nitric oxide formation and repress peroxisome proliferator-activated receptor-γ (PPARγ) signaling [28], which influences calcification. In hypogonadal men, low levels of androgens by sustaining Nitric Oxide Synthase (NOS) activity [29,30], reducing nuclear factor κB ligand (RANKL) signaling [31], and reducing transforming growth factor beta (TGFβ) signaling might reduce calcification [32].

It is established that TGFβ plays a pivotal role in AS. Elevated levels of TGF-β1 are observed in patients with AS, and studies in mice demonstrated a positive correlation between plasma TGF-β1 levels and disease severity [33]. Furthermore, depletion of TGF-β1 in platelets has been shown to attenuate AS progression in a mouse model [34]. Investigating the interplay between androgen levels, AR signaling, and TGF-β expression in human aortic stenosis could provide valuable insights into the sex-based disparities observed in this disease, given that AR has been shown to bind to TGF-β promoter, potentially regulating its expression [16,35]. 

### 2.4. The Role of Estrogen

Increasing evidence is showing that estrogen is a protective hormone against ectopic mineralization. Furthermore, the post-menopausal estrogen treatments might reduce the risk of cardiovascular calcification if it is administered in the first 5 years of menopause; however, other studies suggest that the initiation of estrogen repletion outside of this period may not confer optimal protection. Indeed, it is dependent on time and estrogen dosing regimens, and further investigations are needed to validate the findings.

Estrogen exerts its biological effects through interactions with ERs. In vitro studies showed that estrogen can reduce L-type Ca^2^⁺ channel activity, potentially protecting mice from the detrimental effects of elevated intracellular Ca^2^⁺, contributing to cardiac hypertrophy via calcineurin (Ser/Thr-phosphatase) activation. Notably, 17β-estradiol administration attenuated pressure-overload hypertrophy development in a rat model of aortic stenosis. This protective effect was associated with inhibited phosphorylation of p38 mitogen-activated protein kinases (MAPKs) [36]. In contrast to androgens, estrogens can suppress a variety of molecular processes known to drive cardiovascular calcification, including repression of receptor-activator of nuclear factor-kappa B ligand (RANKL) [37] and nuclear factor-kappa B (NFκB) signaling and suppression of NADPH oxidase activity in resident cells [38].

### 2.5. Sex Hormones and Mitochondrial Activity

Sex hormones can regulate mitochondrial activity, thus directly affecting cellular activity [39]. Androgen affects mitochondrial biogenesis by activating the AR/PGC-1α/TFAM pathway [40], autophagy [41], and ATP production [42]. Similarly, estrogen affects mitochondria phospholipid content of membranes, oxidant and antioxidant capacities, oxidative phosphorylation, and calcium retention capacities [43]. The difference in energy metabolism may significantly shape the outcome of aortic stenosis in men and women.

Finally, although indirect evidence pointing toward a link between sex hormones and AS exists, direct evidence is yet to be established.

## 3. Sex-Related Differences in Cardiac Imaging in Patients with Aortic Stenosis

Many differences have been highlighted between male and female patients with aortic stenosis (AS). These include anatomical and pathophysiological aspects that involve both the valve leaflets and the ventricular remodeling in response to pressure overload, which, in turn, will translate into differences in imaging findings [44,45,46].

### 3.1. Aortic Valve (AV)

Women generally have a smaller AV anatomy, with a smaller aortic annulus and a smaller aortic root. Studies of explanted aortic valves from patients undergoing surgery for AS have shown that men have heavier and more calcified valves than women [47,48]. This difference persisted after adjustment for anatomical factors like the left ventricular outflow tract (LVOT) size and body surface area, regardless of the AV’s morphology (tricuspid vs. bicuspid) [48]. Recent studies using multidetector computed tomography (MDCT) to assess AV calcification also showed that, for the same degree of AS severity, men have more severe AV calcification than women, even after adjustment for the size of the aortic annulus [49]. These findings suggest that a lower calcium scoring should be considered in women to diagnose severe AS [49]. This intriguing difference in calcification patterns between men and women has led to further research studies that suggest that women may have more fibrotic remodeling of AV leaflets than men despite having the same degree of AS severity [47]. This difference may be in part related to the hormonal influence highlighted above.

### 3.2. Left Ventricular (LV) Remodeling

Women are more likely to present with concentric remodeling and a small LV cavity, lower stroke volume despite preserved left ventricular ejection fraction (LVEF), and a more severe diastolic dysfunction due to reduced LV compliance. On the other side of the spectrum, men usually present with eccentric remodeling with less severe diastolic dysfunction [44,46]. According to cardiac magnetic resonance (CMR) studies, women also tended to have higher LVEF than men [50]. CMR studies also showed that women often exhibit lower LV mass compared to men, while data from echocardiographic studies are conflicting [46,51,52].

At the ultrastructural level, studies with advanced imaging techniques like CMR with T1 mapping and data from pathology showed a critical difference between men and women regarding LV remodeling and adaptation to the pressure overload resulting from the AS. Accordingly, women generally have an increased fraction of extracellular volume (ECV), a measure of diffuse myocardial fibrosis. At the same time, men are more likely to present with focal myocardial fibrosis, evidenced by the presence of late gadolinium enhancement (LGE) [50,51]. Diffuse fibrosis (e.g., ECV) is potentially reversible after aortic valve replacement, whereas focal myocardial fibrosis (e.g., LGE) is non-reversible [50,51,53]. The pattern of fibrosis in men with AS appears to be mainly driven by the severity of the AS and the extent of LV hypertrophy. In contrast, it seems to be multifactorial in women [33,45,54,55,56,57]. 

### 3.3. Clinical Presentation

Women are more likely to present with atypical symptoms such as dizziness, fatigue and dyspnea [58]. This can be due to the differences in pathophysiological adaptations in response to pressure overload in men and women. Women present more frequently with concentric remodeling/hypertrophy and the resulting diastolic dysfunction, while men present more with eccentric remodeling. Associated comorbidities may also play a role in the clinical presentation. Indeed, women are more likely to present with dysfunction of the coronary microcirculation with atypical presentation while men have a higher prevalence of coronary artery disease (stenosis) which presents as angina during exercise [58,59]. Furthermore, paradoxical low flow, low gradient AS, which presents with a low stroke volume despite a preserved LVEF and a more advanced stage of LV diastolic dysfunction, is more frequent in women and could play a role in the atypical presentation and additional challenges in the diagnosis and grading of AS severity [45]. 

## 4. Challenges in Imaging for Diagnosis of AS Severity in Men and Women

### 4.1. Echocardiography

**Figure 4:**
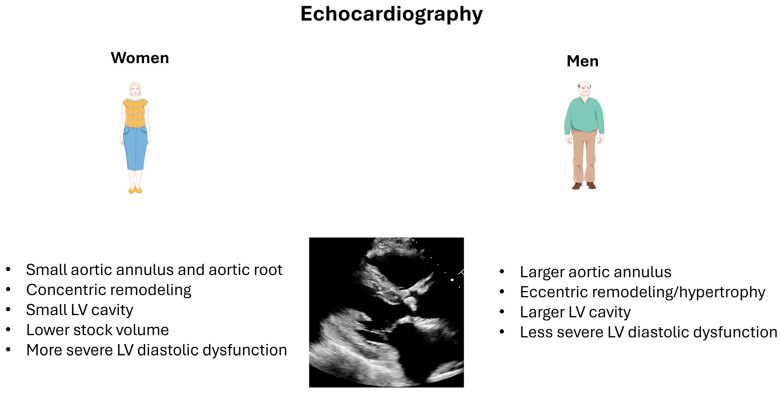
Echocardiographic findings in men and women with aortic stenosis.

Doppler echocardiography is the primary modality for the diagnosis and the assessment of AS severity [60]. However, the accuracy of this modality is limited in circumstances that do not allow appropriate visualization of the AV or the accurate measurement of transvalvular gradients, especially when the Doppler beam is not aligned parallel to the direction of the high-velocity jet [60]. In such circumstances, AS severity may be underestimated, which may cause a delay in diagnosis and late referral for treatment. Furthermore, assessment of AS severity may be very challenging in cases with discordance between aortic valve area (AVA) (being severe, i.e., <1 cm^2^) and gradient (being non-severe; i.e., <40 mmHg) as in patients with paradoxical low-flow low-gradient (LFLG) AS, which is more frequent in women [60,61]. This aspect becomes even more problematic when the leaflets are less calcified despite severe AS, which is more likely to be seen in women [47,48]. This may lead to late diagnosis and late referral for AV replacement in women, especially if the symptoms are atypical (Figure 4).

### 4.2. Cardiac Computed Tomography (CT)

**Figure 5:**
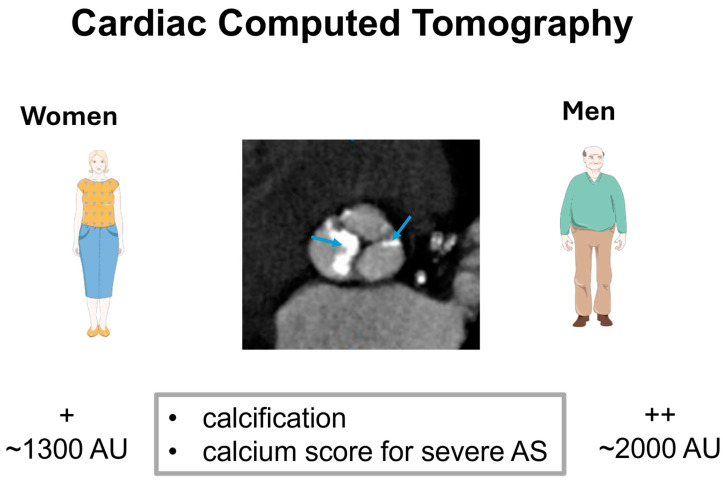
Cardiac computed tomography shows a calcified aortic valve, with a different threshold in men and women for the diagnosis of severe AS. The blue arrows represent the calcification of the aortic valve.

Cardiac CT scans remain helpful for assessing AV calcification in patients with AS when assessment of AS severity by Doppler echocardiography is inconclusive [60]. As highlighted above, studies have shown that women exhibit a less severe calcification than men for the same degree of AS severity, and this difference persists even after AV size is adjusted by the size of the aortic annulus and after adjustment for BSA [47,48,49]. Therefore, current guidelines recommend using different thresholds of calcium scoring in men and women to diagnose severe AS. Accordingly, a calcium score threshold of ~1300 AU is considered sufficient to diagnose severe AS in women, while a score of ~2000 AU is needed to diagnose severe AS in men (Figure 5) [49,60]. However, due to the critical pathophysiological differences of AV remodeling highlighted above, some cases can present with severe AS despite a calcium score lower than the recommended threshold, especially in women, where the correlation between calcification and AS severity and the weight of the explanted valves—a surrogate of AS severity—was less good in women compared to men [47]. Therefore, one should be careful not to rule out severe AS if the valve leaflets exhibit severe thickening with a severely reduced opening (AVA < 1cm^2^) despite a low mean transvalvular gradient (MG < 40 mmHg). This is also true in cases of AS due to or associated with amyloidosis, where AS can be severe despite less severe calcifications due to a more fibrotic remodeling of the valve and the direct infiltration by the amyloidosis process [62]. In these challenging circumstances, further studies, including stress echocardiography when indicated and possible (exercise echo with preserved ejection fraction (EF) and dobutamine stress echocardiogram (DSE) with reduced EF) or, rarely, invasive assessment, are usually needed to confirm AS severity [60].

## 5. Clinical Outcome after Aortic Valve Replacement

The prognosis of AS without treatment is poor [63,64]. The only available treatment is aortic valve replacement (AVR), which can be performed either through surgery (SAVR) or transcatheter aortic valve replacement (TAVR) [65,66,67]. Recent studies have shown that the 5-year survival after diagnosis of AS was worse in women than in men, and the difference was attributed to a more conservative management of AS in women [68]. Accordingly, women appear to be less likely to be referred for AVR than men, with a later referral, older age, and more advanced stage of the disease at presentation [68,69]. This late referral may be due to multiple reasons, such as an atypical clinical presentation, which may lead to a late diagnosis, older age at presentation, or a more challenging diagnosis of AS severity, as highlighted above. 

Selecting the optimal therapy for each patient depends on several factors, including the patient’s anatomy and risk profile, valve durability, and patient preferences [70]. 

### 5.1. Transcatheter Aortic Valve Replacement (TAVR)

After both TAVR and SAVR, women appear to have less severe myocardial fibrosis, more favorable left ventricular remodeling, and faster regression of LV hypertrophy than men [71,72].

Overall, among patients who underwent TAVR for severe AS, studies showed no significant difference in 30-day mortality rates between men and women [73,74,75] despite a greater rate of procedural-related complications in women, like bleeding, vascular complications, and conversion to SAVR [73,76,77,78,79,80]. These complications are, at least in part, a direct consequence of the smaller aortic valve annulus and vascular anatomy and higher rates of porcelain aorta in women than men [81,82]. Recent studies also showed a link between the type of transcatheter valve and the rate of complications, with more common complications in women treated with self-expanding valves [83]. This trend was mainly driven by vascular complications [83].

Several studies, including a recent metanalysis of more than forty-seven thousand patients, showed that despite older age and higher risk profile at the time of TAVR and a higher rate of short-term complications like severe bleeding and vascular complications, women had similar or even better survival compared to men [73,84,85,86,87]. This is referred to by some scientists as ‘’the women paradox’’. It is worth mentioning though that several baseline factors, but not sex, were predictors of mortality [87].

Although data from previous studies suggest that women may have a more significant benefit from TAVR over SAVR during follow-up [58,73,75,76,77,78,88,89], these data are not based on sex-based randomization. One explanation for the potential benefit of TAVR in women as compared to men is thought to be related to a lower prevalence of significant paravalvular regurgitation (PVR)—a powerful prognostic marker following TAVR—as a direct result of less severe AV calcification and a smaller AV annulus size [90]. The presence of PVR can be more problematic in the setting of small LV size and restrictive physiology. This pattern is more observed in women, where even a less severe but acute PVR can have detrimental hemodynamic consequences. This benefit of TAVR over SAVR in women could also be explained by a lower rate of prosthesis–patient mismatch (PPM) following TAVR as compared to SAVR [91] with a more favorable LV reverse remodeling in women after TAVR [73]. The RHEIA trial is a recent prospective, randomized trial that was designed to investigate whether TAVR is superior to SAVR in female patients with severe symptomatic AS, regardless of the surgical risk [92]. The preliminary results of this study presented at the ESC meeting in August 2024 suggest that TAVR was superior to SAVR for reducing death, stroke or rehospitalization, and the benefit was mostly driven by a lower rate of rehospitalizations. TAVR was also associated with a shorter hospital stay compared to SAVR. These data suggest that TAVR could be considered the preferred option to treat women with severe aortic stenosis.

There are some conflicting data regarding pacemaker implantation rates following TAVR [93]. However, a large meta-analysis suggests that women have a lower risk of permanent pacemaker implantation after TAVR [94]. This can also explain the greater benefit of TAVR in women. 

However, it is important to remember that long-term data regarding the durability of transcatheter valves are still limited, and further studies are warranted. 

### 5.2. SAVR

Being a biological woman is a prognostic surgical risk factor per se, and it is included in risk scores like the STS score [95]. Although women are at higher risk of complications after surgical AVR [95,96,97,98], some studies have shown that despite older age and more comorbidities, women did not have increased postoperative and long-term mortality after SAVR [96]. However, data from other studies suggest that women undergoing SAVR have a worse 30-day mortality risk compared to men [69,99]. This could be explained by the higher rate in women of vascular complications, bleeding, and blood transfusions with their negative consequences [88]. In addition to a higher rate of prosthesis–patient mismatch due to a smaller aortic annulus in women, and the associated comorbidities like renal and heart failure which makes the prognosis less favorable [74]. Indeed, moderate and severe PPMs are more frequent in women after SAVR and were associated with increased in-hospital mortality as compared to those with no mismatch [100]. PPM was also associated with poorer long-term outcomes, including higher all-cause and cardiac death, heart failure, and rehospitalization [101,102,103,104], highlighting the need for the implementation of preventive strategies to avoid PPM after SAVR. 

## 6. Conclusions

In the present review, we discussed the potential sex-specific molecular mechanisms and clinical presentation and outcomes in male and female patients with aortic stenosis. Although important differences between men and women with AS have been highlighted, understanding the underlying mechanisms leading to these differences is still limited. Further studies on the pathophysiology and outcomes of AS in male and female patients are warranted to understand these differences better, personalize the disease’s management, and improve the outcomes.
